# Diagnostic accuracy of mercurial versus digital blood pressure measurement devices: a systematic review and meta-analysis

**DOI:** 10.1038/s41598-022-07315-z

**Published:** 2022-03-01

**Authors:** Malaisamy Muniyandi, Senthil Sellappan, Vidya Chellaswamy, Karunya Ravi, Sananthya Karthikeyan, Kannan Thiruvengadam, Jerard Maria Selvam, Nagarajan Karikalan

**Affiliations:** 1 Department of Health Economics, ICMR-National Institute for Research in Tuberculosis, No. 1, Sathyamoorthy Road, Chetpet, Chennai, 600031 India; 2 ICMR-Regional Medical Research Centre, Port Blair, Andaman and the Nicobar Islands India; 3 Department of Public Health & Preventive Medicine, Government of Tamil Nadu, Chennai, India

**Keywords:** Cardiology, Health care, Medical research, Translational research

## Abstract

This study aims to systematically review the diagnostic accuracy of a digital blood pressure measurement device compared to the gold standard mercury sphygmomanometer in published studies. Searches were conducted in PubMed, Cochrane, EBSCO, EMBASE and Google Scholar host databases using the specific search strategy and filters from 1st January 2000 to 3rd April 2021. We included studies reporting data on the sensitivity or specificity of blood pressure measured by digital devices and mercury sphygmomanometer used as the reference standard. Studies conducted among children, special populations, and specific disease groups were excluded. We considered published manuscripts in the English language only. The risk of bias and applicability concerns were assessed based on the author’s judgment using the QUADAS2 manual measurement evaluation tool. Based on the screening, four studies were included in the final analysis. Sensitivity, specificity, diagnostic odds ratio (DOR), and 95% confidence interval were estimated. The digital blood pressure monitoring has a moderate level of accuracy and the device can correctly distinguish hypertension with a pooled estimate sensitivity of 65.7% and specificity of 95.9%. After removing one study, which had very low sensitivity and very high specificity, the pooled sensitivity estimate was 79%, and the specificity was 91%. The meta-analysis of DOR suggests that the digital blood pressure monitor had moderate accuracy with a mercury sphygmomanometer. This will provide the clinician and patients with accurate information on blood pressure with which diagnostic and treatment decisions could be made.

## Introduction

Hypertension is one of the major chronic diseases that leads to higher mortality worldwide. Early diagnosis and treatment of hypertension could significantly reduce the risk of mortality^1^. A mercury sphygmomanometer is a standard instrument that is used manually to monitor blood pressure in health care facilities. This method is considered the gold standard and has been used in practice for more than a hundred years^2^. However, mercury-based devices have limitations due to environmental contamination and observer bias in measurement. To address these limitations, wearable and portable digital devices that could monitor blood pressure were introduced and used by health care professionals and patients^3,4^. Multiple guidelines are available to appropriately use digital devices for self-monitoring blood pressure by patients at their convenience and in health facilities^5,6^. However, the convenience and flexibility of these automated digital devices should be considered with caution about their measurement accuracy^5^.

The benefits and efficiency of blood pressure screening methods require an evidence base about measurement accuracy and validity^7^. Multiple validation studies have been undertaken to assess the importance of the diagnostic accuracy of mercury-based and digital devices for blood pressure monitoring. However, these validation studies need to be considered with caution concerning the quality of evidence and standard of method that they have used. At present, there is a lack of systematic reviews on the diagnostic accuracy of digital blood pressure measurement devices. In this background, we undertook a systematic review to generate evidence on the diagnostic accuracy of a digital blood pressure measurement device compared to the gold standard mercury sphygmomanometer^8^.

## Methods

This systematic review was conducted based on the Preferred Reporting Items for a Systematic Review and Meta-analysis of Diagnostic Test Accuracy Studies (PRISMA-DTA)^9^ (Supplementary Appendix). The protocol for this systematic review was registered on PROSPERO the International Prospective Register of Systematic Reviews (Registration number CRD42019118822).

### Information sources

A comprehensive literature search was undertaken in search engines that included PubMed, Cochrane, EBSCO, EMBASE and Google Scholar.

### Search strategy

The search terms used to retrieve published information specifically for each database are provided in Supplementary Table A1. The number of articles obtained and further filtering based on eligibility criteria are provided in Table A1.

### PICO elements

#### The PICO

The PICO criteria for the systematic review are given in Supplementary Table A2.

#### Population

Studies done in more than 18 years of age group.

#### Intervention test

Digital blood pressure monitoring devices.

#### Comparator test

A mercury sphygmomanometer was used manually.

#### Outcomes

The sensitivity and specificity of digital and mercury sphygmomanometer-based blood pressure measurements.

### Eligibility criteria

#### Types of studies

Cross-sectional and observational cohort studies assessing the diagnostic accuracy of blood pressure measured by mercury sphygmomanometer and digital devices.

#### Language and time period

Journal articles published in English between 1st January 2000 and 3rd April 2021 with full-text accessibility were included.

#### Reference standard

A mercury sphygmomanometer was used as the reference standard.

### Exclusion criteria

Diagnostic accuracy studies that considered digital blood pressure monitoring devices using a standard nonmercury sphygmomanometer as a comparator, and studies conducted among children, special populations and specific disease groups were excluded.

### Study selection process

The studies were screened individually by two reviewers, and any discrepancies raised in the selection process were resolved with the third reviewer. Duplicates were removed from the studies shortlisted after the title and abstract screening. Full-paper screening was performed for the shortlisted studies. Studies that did not meet the selection criteria did not have relevant information for inclusion and that required data were excluded. The remaining eligible studies were included in the review.

### Quality and risk assessment

The quality of the included studies was assessed using the QUADAS-2 questionnaire (Table A3 in Supplementary)^10^, a standard tool used for quality assessment of diagnostic accuracy studies. This questionnaire measures the risk of bias and applicability concerns.

### Index test and standard

The index test studied was a digital blood pressure monitor, which is a cuffed device used to measure blood pressure by self or by trained staff. The threshold used for hypertension or high blood pressure was ≥ 140 for systolic blood pressure and ≥ 90 for diastolic blood pressure or as defined by the included studies. A mercury sphygmomanometer was the reference standard test used.

### Data extraction

The data were extracted from the included studies using the data extraction form. We collected information on the screening instrument used, the reference standard employed, indices of diagnostic accuracy, statistical and methodological considerations. Data on study characteristics such as year, settings, population, design, comparator, and sample size were also collected.

### Data synthesis and analysis

The data collected were entered into the Microsoft Excel worksheet. We qualitatively described the characteristics of the studies included in the review. Indices of diagnostic accuracy, including true-positive, true-negative, false-positive, false-negative, positive predictive value, negative predictive value, positive likelihood ratio, negative likelihood ratio, sensitivity and specificity were calculated. The diagnostic odds ratio (DOR) and 95% confidence interval were estimated. For the quantitative meta-analysis, RevMan software (version 5.4) and R studio were used. A forest plot was used to depict the pooled estimates for DOR, sensitivity, and specificity. The heterogeneity of the included studies was also assessed by the *I*^2^ statistic for all the parameters assessed.

## Results

### Study selection

**Figure 1:**
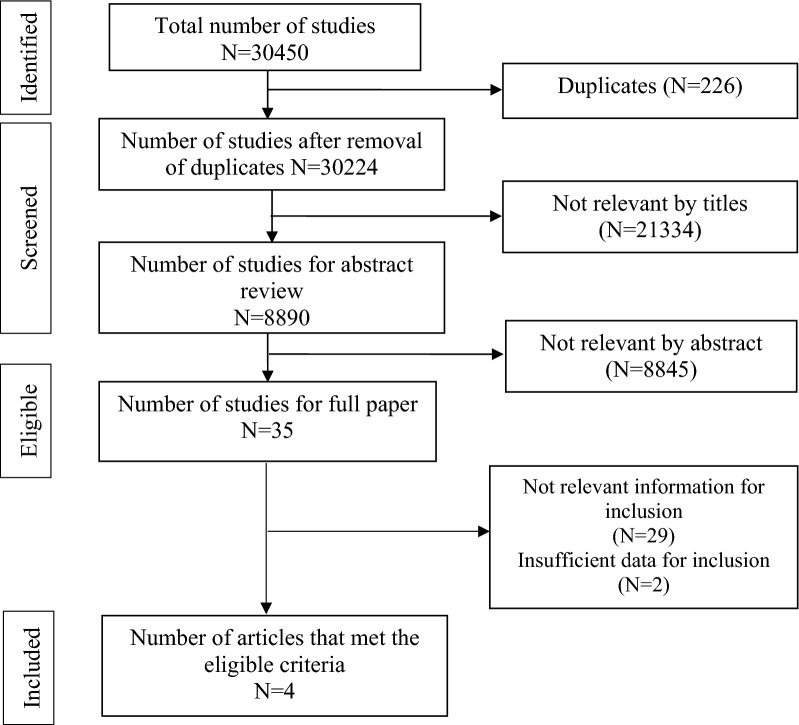
PRISMA flow diagram indicating the process of selecting the study.

The PRISMA flowchart describes the study selection process (Fig. 1). Overall, 30,450 articles were initially identified in our search; of these, 226 duplicates were removed, and 30,224 article titles and abstracts were screened. Studies with potentially relevant titles or abstracts were subjected to full-text screening based on the eligibility criteria. Based on the screening, four studies were included in the final analysis.

### Characteristics of the studies included

The general characteristics of the included studies are presented in Table 1. The articles were published between 2010 and 2021. Two studies were conducted in India^11^, while the other two were conducted in Colombia and the USA. Of these studies, two studies were conducted among the general population, one at the community health center and one among nursing college students. The cohort study design was adapted in three studies, while one study was cross-sectional in design. All studies used a mercury sphygmomanometer as the reference standard with systolic and diastolic blood pressure cut-offs at 140 and 90 respectively. The sample size ranged from 108 to 1084, with the total study population accounting for 1919. The average age of the study population in these studies ranged from 40 to 54 years. The average systolic blood pressure reported in the included studies varied from 107.10 to 172 mmHg. Similarly, the average diastolic blood pressure reported varied from 67.98 to 96.3 mmHg.

**Table 1:** Characteristics of the studies included in the evidence review.

Author and Year	Country	Settings	Population	Design	Sample	Comparison	Conclusion
Ostchega et al. (2010)^2^	USA	National Health and Nutrition Examination Survey (NHANES)	Individuals aged ≥ 13 years	Longitudinal survey	509	Mercury Sphygmomanometer vs Digital blood pressure monitor (Omron HEM-907XL)	Digital blood pressure monitor tends to underestimate the prevalence of hypertension as measured by HgS by 2.65%
Vera-Cala et al. (2011)^28^	Colombia	Epidemiological study	Individuals aged 15–64 year	Cohort	1084	Mercury sphygmomanometer vs Automatic device (Omron HEM-705-CP)	Omron HEM-705-CP could be used for measuring blood pressure in large epidemiology studies without compromising precision
Bhatt et al. (2016)^11^	India	Nursing College Student	Nursing student age ≥ 18 years	Cohort	108	Mercury sphygmomanometer vs Digital device	The sensitivity of the digital sphygmomanometer was found unsatisfactory
Shahbabu et al. (2016)^12^	India	Community-based Health Centre	Individuals aged ≥ 25 years	Cross-sectional	218	Mercury sphygmomanometer (NOVAPHON) vs Digital device (Omron Hem-7111)	The digital device had less accuracy. Sensitivity and specificity of a digital device (80% and 67.7%)

### Risk of bias and applicability concern assessment

**Figure 2:**
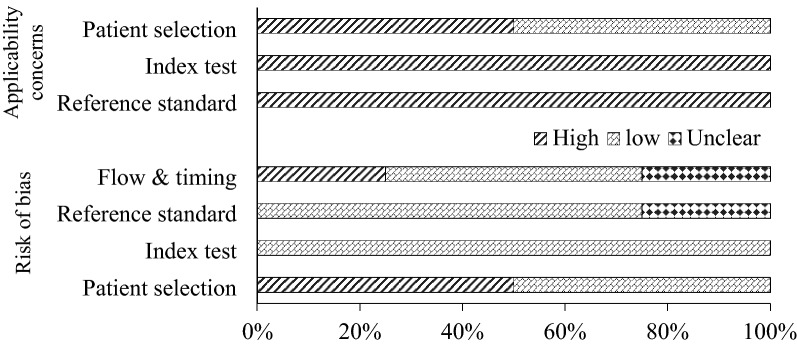
Risk of bias and applicability-concerns graph presenting authors judgements based on QUADAS2 evaluation tool.

The risk of bias assessment was done by two reviewers and applicability concerns were assessed based on the author’s judgment using the QUADAS2 evaluation tool (Fig. 2). Of the studies included, 50% and 25% reported a high risk of bias in patient selection, flow and timing domains respectively. The risk of bias was low in all included studies for the index test domain and 50% of the studies reported a low risk of applicability concern in patient selection.

### Quantitative data synthesis

The measures of diagnostic accuracy for digital blood pressure monitoring, including true-positive, true-negative, positive predictive value, negative predictive value, sensitivity and specificity estimated in each study are presented in Table 2. The positive predictive value of the included studies was close to 1 (0.96–1), except for a clinic-based study, which had a positive predictive value of 0.48.

**Table 2:** Summary of diagnostic accuracy measures for blood pressure monitoring in the studies included in the evidence review.

Study	Sample	TP	FN	TN	FP	PPV	NPV	LR+	LR−	Sensitivity	Specificity
Ostchega et al. (2010)^2^	509	204	97	200	8	0.962	0.673	16.67	0.34	67.86	95.93
Vera-Cala et al. (2011)^28^	1084	693	93	294	4	0.994	0.76	63	0.12	88.20	98.6
Bhatt et al. (2016)^11^	108	4	30	74	0	1	0.71	101	0.89	11	100
Shahbabu et al. (2016)^12^	218	48	12	107	51	0.485	0.899	2.48	0.3	80.0	67.7

*TP* true positive, *FN* false negative, *TN* true negative, *FP* false positive, *PPV* positive predicted value, *NPV* negative predicted value, *LR*+ likelihood ratio positive, *LR−* likelihood ratio negative.

**Figure 3:**
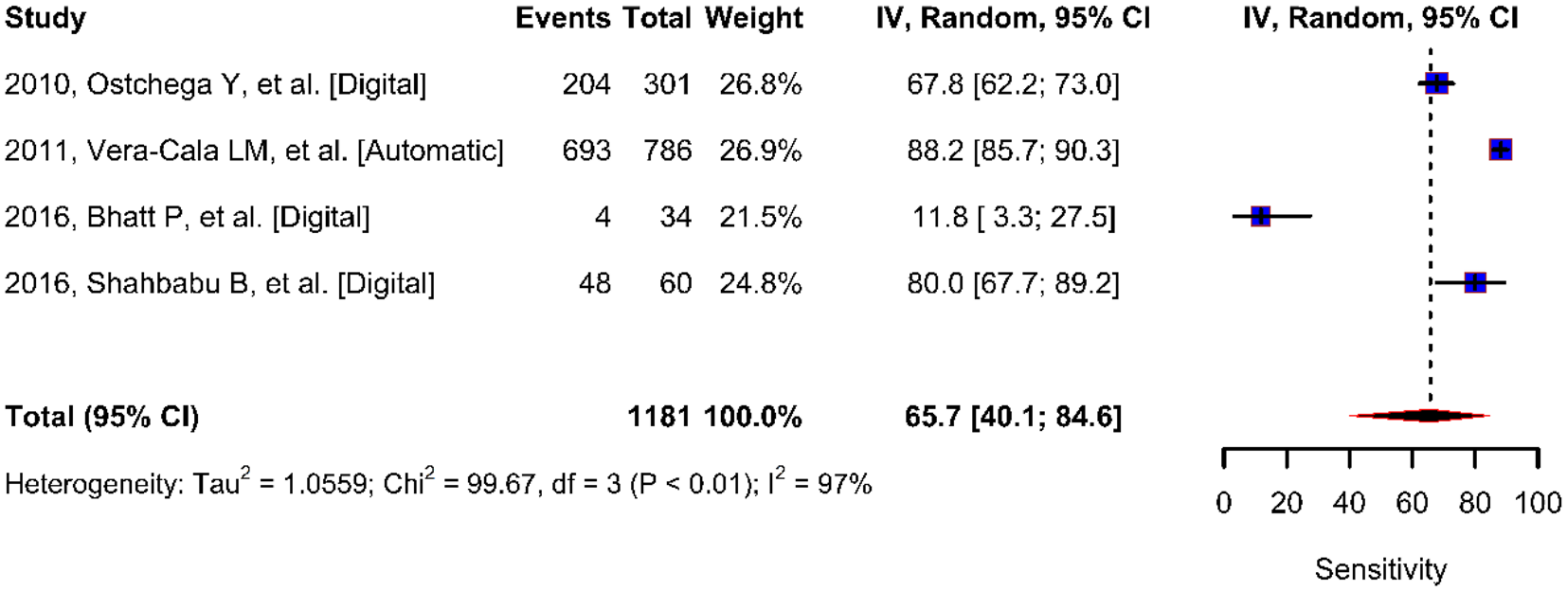
Forest plot for digital blood pressure monitor true positive rate (sensitivity).

**Figure 4:**
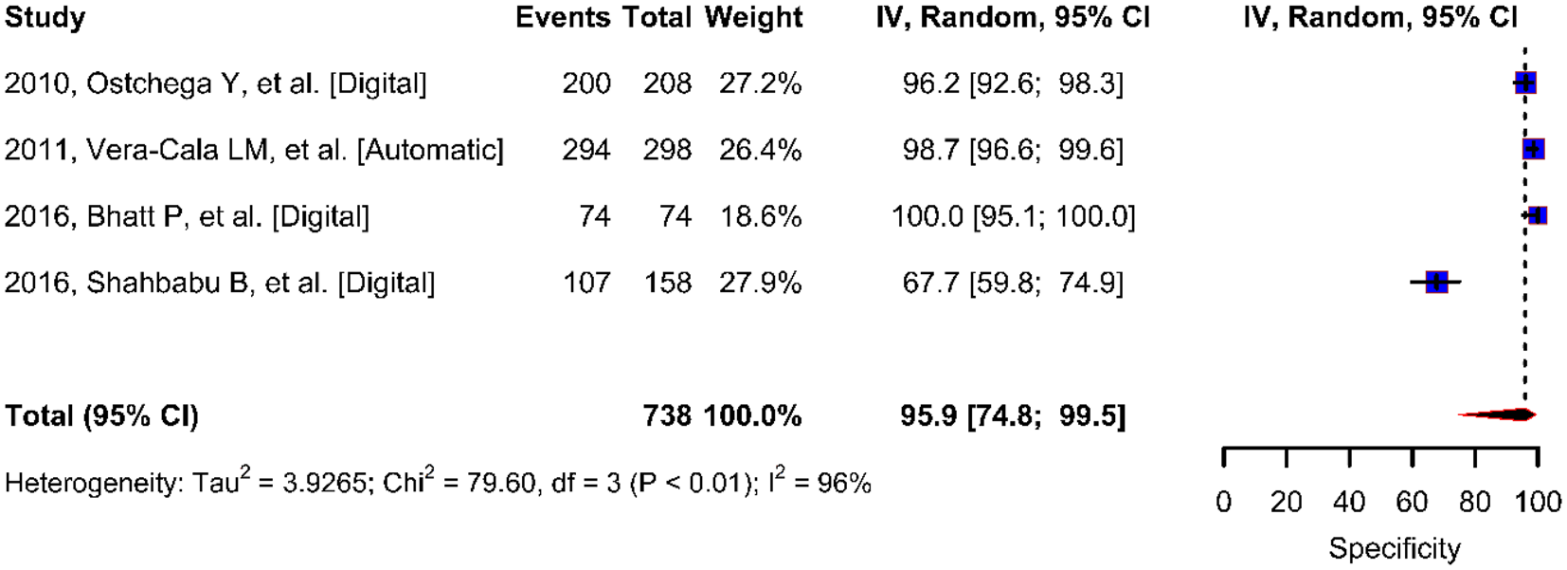
Forest plot for digital blood pressure monitor true negative rate (specificity).

The sensitivity of the digital blood pressure monitor given by the proportion of test positives in the subjects with disease ranged from 12 to 88%, and the pooled estimate was 64% (95% CI 40–85) (Fig. 3). The specificity of the digital blood pressure monitor given by the proportion of test negatives in the subjects without disease ranged from 68 to 100%, and the pooled estimate was 94% (95% CI 79–100) (Fig. 4). There was significant heterogeneity in sensitivity and specificity in these studies. Therefore, we also repeated the analysis by removing very low sensitivity and very high specificity resulting in a pooled sensitivity estimate of 79% and specificity of 91%.

**Figure 5:**
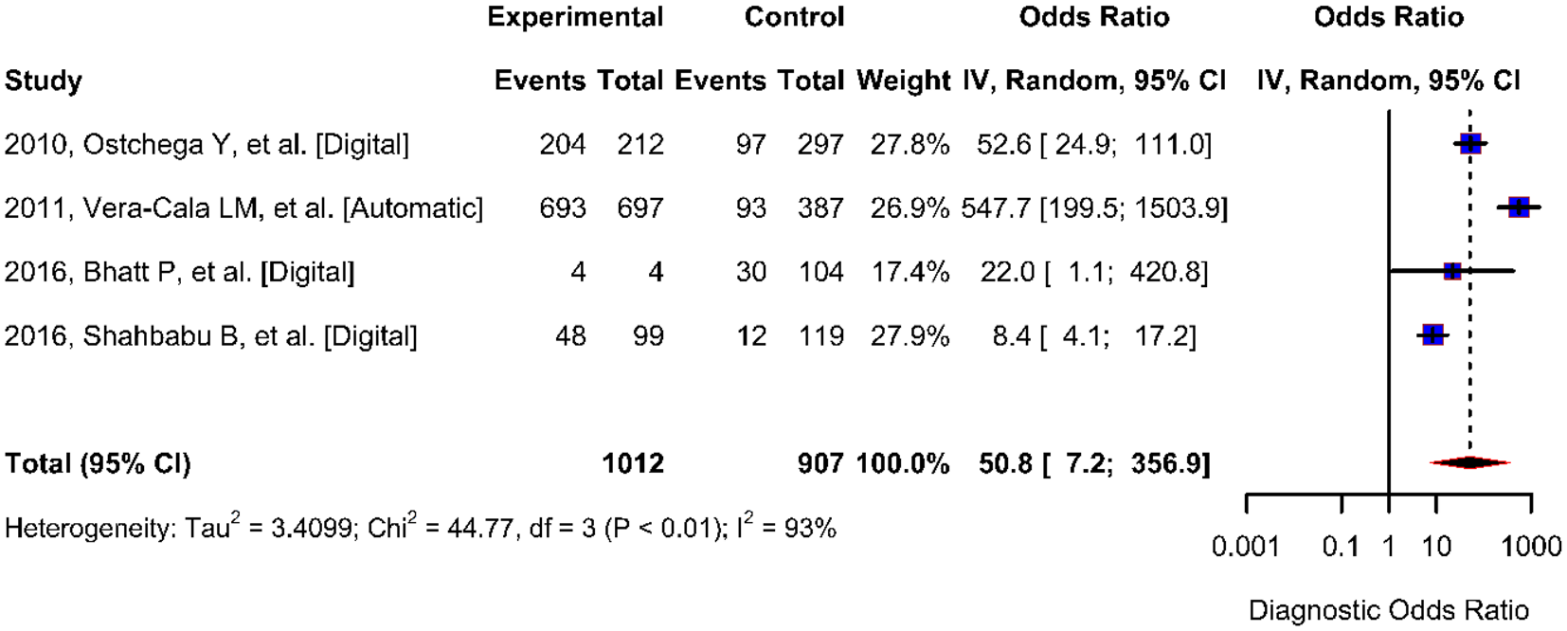
Forest plot of diagnostic odds ratio of digital blood pressure monitor.

**Figure 6:**
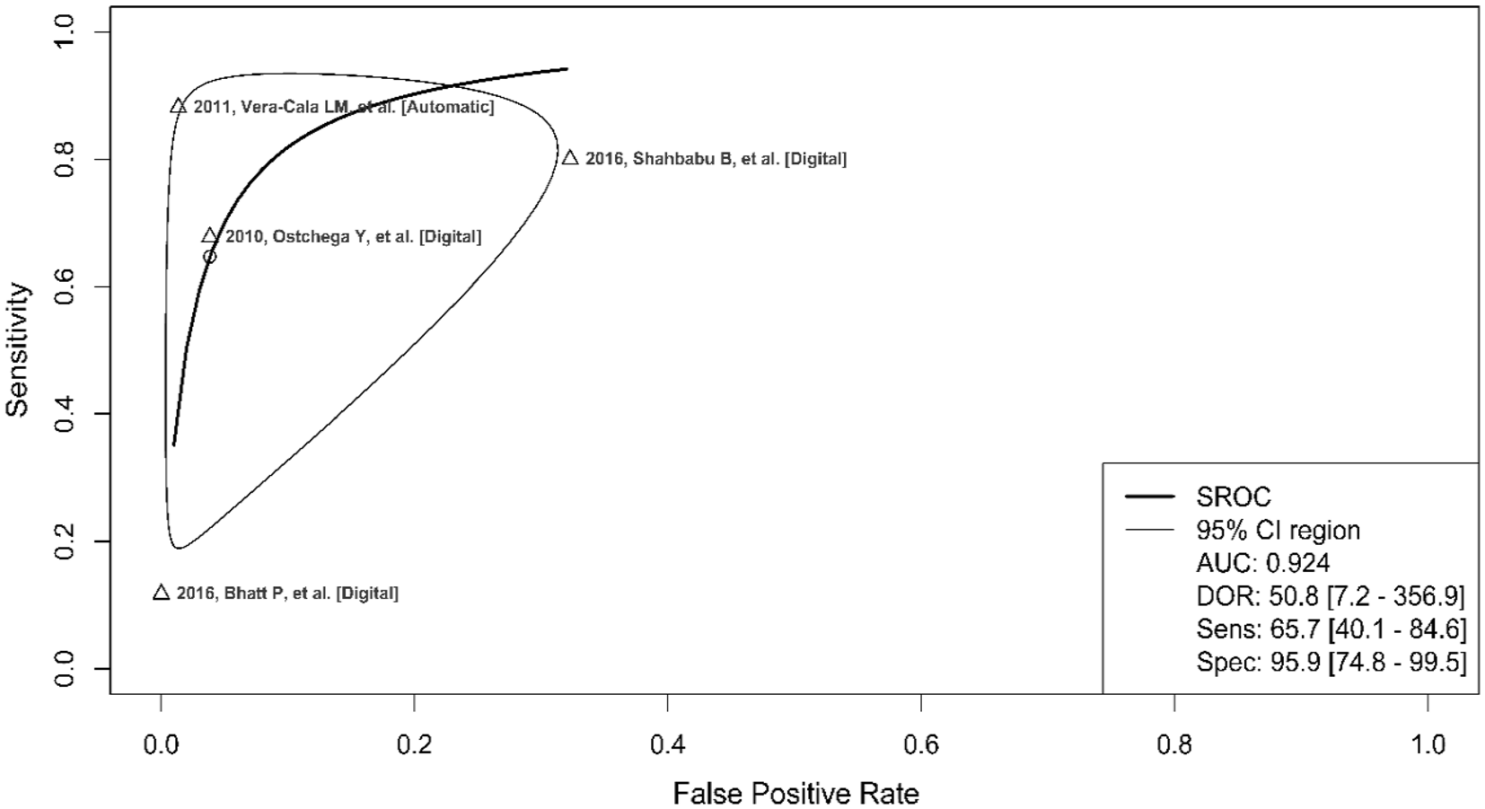
Summary receiver operating characteristic (SROC) curve for digital blood pressure monitor diagnostic test accuracy.

The odds of obtaining a positive test result in a diseased individual compared to a non-diseased individual (DOR) using a digital blood pressure monitor ranged from 8.39 to 547.69 with a pooled estimate of 50.84 (95% CI 7.24–356.87) (Fig. 5). The DOR estimated in studies conducted at health facilities was lower (8.39, 21.98) than that estimated in studies conducted in the community (52.58, 547.6). Heterogeneity presented as *I*^2^ values were considerably higher than 50 in all the parameters assessed. The receiver operating characteristic (ROC) curve points represent different studies, and the fitted summary ROC curves depict trade-offs between sensitivity and specificity that arise because of differences between the studies (Fig. 6).

## Discussion

This meta-analysis evaluated the studies concerning the accuracy of a digital blood pressure measurement device compared to the gold standard mercury sphygmomanometer to diagnose hypertension. Rapid early diagnosis is essential to control hypertension and cardiovascular diseases. In the current meta-analysis, the diagnostic reference standard used was similar in all included studies. The meta-analysis results of DOR suggest that digital blood pressure monitoring has a moderate level of accuracy and that the device can correctly distinguish hypertension with a pooled estimated sensitivity of 65.7% and specificity of 95.9%. After removing one study, which had very low sensitivity and very high specificity, the pooled estimates of sensitivity were 79% and specificity of 91%, respectively. Our analysis suggests that digital blood pressure monitoring had equal accuracy with mercury sphygmomanometer. Our findings corroborate the studies that reported that digital blood pressure monitoring should be used for proper and better management of hypertension^12,13^. We reemphasize the fact that for correct estimation of blood pressure, mercury sphygmomanometers are considered the gold standard, with the comparability of measurement accuracy, found that digital blood pressure monitoring is almost as accurate as mercury sphygmomanometer. Digital monitoring can be substituted with the traditional mercury sphygmomanometer.

High blood pressure is a major cardiovascular risk factor that is treatable; however, knowledge, attitudes and practices remain low. Digital blood pressure measurement tools could improve the management of hypertension by enabling individual monitoring of blood pressure anytime and anywhere^14^. It is also useful in continuous monitoring of measurements of blood pressure from the mass population during daily life^15^. The use of mercury devices for blood pressure measurement continues to diminish their role and the preferable modality of blood pressure measurement has changed in health systems. Mercury devices have placed new interest in alternative methods, of which digital devices are the leading nominees. However, the error reported on the accuracy of digital devices ranged from 1 to 44%^16,17^. Validation studies that compared mercury versus digital devices in the setting of a large clinical trial found it to be accurate^18^.

The effect of potential mercury toxicity and the problems associated with the disposal of mercury has led to widely reduced usage of mercury instruments across the world. For this reason, the European Union recently directed phasing out of mercury instruments. The advantages of digital blood pressure monitoring are that it is easy to use, more affordable, portable, and has no adverse environmental impacts. A large study on the accuracy of digital instruments reported that it was almost as accurate as mercury instruments^19,20^. In this respect, there is a paucity of information in the Indian context. Intensive blood pressure control has been found to significantly reduce cardiovascular morbidity and all-cause mortality associated with hypertension^21^. Regular and frequent measurement of blood pressure was recommended for a patient with hypertension to prevent cardiovascular morbidity^22,23^. The technology revolution and development of new technologies allow people to use and would enhance autonomy while providing physicians with a more complete picture of their blood pressure profile, leading to blood pressure control and better long-term clinical outcomes^24^.

There is a need to consider the disadvantages associated with digital blood pleasure monitoring devices in terms of lifespan and quality standards^25^. Considering all this, there is a need to utilize more feasible and inexpensive instruments without frequent replacements, particularly for low-income countries.

The current DOR estimated in studies conducted at health facilities was lower than that estimated in studies conducted in the community. This underscores that the device with high specificity was found to be better performing in community-based studies than in a health facility setup. This may be due to the better performance device in a large population. Control of blood pressure begins with an accurate measurement, leading to appropriate diagnosis and treatment^26^. The digital blood pleasure monitor is a simple, accurate and affordable device that can be used under field conditions in the management of hypertension. In the advanced digital health information technology era, there is a paradigm shift from traditional methods to modern methods. These methods are expected to change the quality of detection of hypertension and management. This new approach contributes to early diagnosis and prevention, which refers to identifying risk and predicting the onset of cardiovascular events and allowing interventions to reduce risk. India is home to an estimated 234 million adults who have hypertension^27^. Like diabetes patients who have glucometers at home, asthma patients who have a peak flow meter at home, pyrexia patients who have a thermometer at home and COVID patients who have a pulse oximeter at home, we may also find blood pressure monitoring in every patient’s home.

We are interpreting our meta-analysis results with few limitations. First, potential heterogeneity was explored in the included studies in our meta-analysis. The second was that number of studies is limited. The present study identified only four studies which met our inclusion criteria. Of this two studies were conducted in general population and thus more generalizable, but other two studies were conducted in institutional setting which might be different from general population. One study was used cross sectional design and rest were cohort design. While this could be a limitation still these studies were meeting the inclusion criteria based on intervention and comparator.

The evidence from this review suggests that digital blood pressure measures were able to correctly rule out hypertension due to their high specificity but have moderate accuracy in identifying true positive cases. It can be utilized for screening a larger population in an efficient and timebound way.

## Supplementary Information

Supplementary Information.

## Data Availability

The original contributions generated for the study are included in the article/supplementary material, and further inquiries can be directed to the corresponding author.
