# Supplementary material for: Diagnostic accuracy of mercurial versus digital blood pressure measurement devices: a systematic review and meta-analysis
Source: Sci Rep. 2022 Mar 1;12:3363. doi: 10.1038/s41598-022-07315-z (PMC8888622; doi:10.1038/s41598-022-07315-z)
Supplement: Supplementary file 1 — Supplementary Information. [file 41598_2022_7315_MOESM1_ESM.docx]

**Appendix**

**Table A1. Systematic review on diagnostic accuracy of various diagnostic tools to screen hypertension**

| **Databases** | **Search Strategies** | **Results** |
| --- | --- | --- |
| **Pubmed** | 1. Mercury free Sphygmomanometer OR clinic blood pressure measurement OR ambulatory blood pressure measurement OR home blood pressure measurement OR clinic blood pressure monitoring OR ambulatory blood pressure monitoring OR home blood pressure monitoring OR Electronic blood pressure measurement OR Electronic pressure transducer OR Oscillometric automated instrument OR Mercury Sphygmomanometer OR Aneroid Sphygmomanometer OR non-mercury devices 2. Blood Pressure OR Hypertension OR blood pressure OR bp OR abp OR hypertens* 3. 1 AND 2 4. blood pressure determination OR Sphygmomanometers OR Sphygmomanometer* OR bp OR abp OR "blood pressure" OR hypertens* AND near/5 device* OR monitor* OR meter* OR metre* 5. 3 OR 4 6. "reproducibility of results" OR "Sensitivity and Specificity" OR calibrat* OR accura* OR error* OR inaccura* OR reliab* OR unreliab* OR valid* OR invalid* OR reproducib* OR standard deviation OR precis* 7. 5 AND 6   # Filter Applied: Comparative Study[ptyp] OR Evaluation Studies[ptyp] OR Validation Studies[ptyp] AND "2000/01/01"[PDat]: "2019/04/03"[PDat] AND Humans[Mesh] AND English[lang] AND Adult: 19+ years | 50421  1126881  49555  998534  1018100  2,795,684  226545  5358 |
| **Cochrane** | #1 (Blood Pressure)  #2 (Hypertension)  #3 (blood pressure or bp or abp or hypertens*)  #4 #1 OR #2 OR #3  #5 (blood pressure determination)  #6 (Sphygmomanometers)  #7 (Sphygmomanometer*)  #8 (bp or abp or "blood pressure" or hypertens* near/5 device* or monitor* or meter* or metre*)  #9 #5 OR #6 OR #7 OR #8  #10 (Mercury free Sphygmomanometer OR clinic blood pressure measurement OR ambulatory blood pressure measurement OR home blood pressure measurement OR clinic blood pressure monitoring OR ambulatory blood pressure monitoring OR home blood pressure monitoring OR Electronic blood pressure measurement OR Electronic pressure transducer OR Oscillometric automated instrument OR Mercury Sphygmomanometer OR Aneroid Sphygmomanometer OR non-mercury devices)  #11 ("reproducibility of results")  #12 ("Sensitivity and Specificity")  #13 (calibrat* or accura* or error* or inaccura* or reliab* or unreliab* or valid* or invalid* or reproducib* or standard deviation or precis*)  #14 #11 OR #12 OR #13  #15 #10 AND #4  #16 #15 OR #14  # Cochrane Reviews  # 1^st^ Jan 2000 to 9^th^ June 2020 | 104047  63174  143727  143727  2961  133  1411  184169  184632  8434  10771  13640  141865  146856  8390  152250  21250 |
| **EMBSCOhost** | S1 - Blood Pressure OR Hypertension OR blood pressure OR bp OR abp OR hypertens*  S2 - Mercury free Sphygmomanometer OR clinic blood pressure measurement OR ambulatory blood pressure measurement OR home blood pressure measurement OR clinic blood pressure monitoring OR ambulatory blood pressure monitoring OR home blood pressure monitoring OR Electronic blood pressure measurement OR Electronic pressure transducer OR Oscillometric automated instrument OR Mercury Sphygmomanometer OR Aneroid Sphygmomanometer OR non-mercury devices  S3- S1 AND S2  S4- Blood pressure determination OR Sphygmomanometers OR Sphygmomanometer* OR bp OR abp OR "blood pressure" OR hypertens* AND near/5 device* OR monitor* OR meter* OR metre*  S5 - "reproducibility of results" OR "Sensitivity and Specificity" ORcalibrat* OR accura* OR error* OR inaccura* OR reliab* OR unreliab* OR valid* OR invalid* OR reproducib* OR standard deviation OR precis*  S6 - S4 AND S5  S7 - S3 OR S6  # Academic Journals AND Journals  # 1^st^ Jan 2000 to 3^rd^ Apr 2019 | 10,808  35  35  56,123  1,86,507  13,149  13,176  1821  1643 |
| **Embase** | 1. ('mercury free sphygmomanometer':ab,ti OR 'clinic blood pressure measurement':ab,ti OR 'ambulatory blood pressure measurement':ab,ti OR 'home blood pressure measurement':ab,ti OR 'clinic blood pressure monitoring':ab,ti OR 'ambulatory blood pressure monitoring':ab,ti OR 'home blood pressure monitoring':ab,ti OR 'electronic blood pressure measurement':ab,ti OR 'electronic pressure transducer':ab,ti OR 'oscillometric automated instrument':ab,ti OR 'mercury sphygmomanometer':ab,ti OR 'aneroid sphygmomanometer':ab,ti OR 'non-mercury devices':ab,ti) AND reproducibility AND of AND results OR (sensitivity AND specificity) 2. #1 AND (2000:py OR 2001:py OR 2002:py OR 2003:py OR 2004:py OR 2005:py OR 2006:py OR 2007:py OR 2008:py OR 2009:py OR 2010:py OR 2011:py OR 2012:py OR 2013:py OR 2014:py OR 2015:py OR 2016:py OR 2017:py OR 2018:py OR 2019:py OR 2020:py OR 2021:py)   #1 AND (2000:py OR 2001:py OR 2002:py OR 2003:py OR 2004:py OR 2005:py OR 2006:py OR 2007:py OR 2008:py OR 2009:py OR 2010:py OR 2011:py OR 2012:py OR 2013:py OR 2014:py OR 2015:py OR 2016:py OR 2017:py OR 2018:py OR 2019:py OR 2020:py OR 2021:py) AND 'hypertension'/dm AND ('cohort analysis'/de OR 'comparative effectiveness'/de OR 'comparative study'/de OR 'cross sectional study'/de OR 'meta analysis'/de OR 'observational study'/de OR 'systematic review'/de) AND ([adult]/lim OR [aged]/lim OR [very elderly]/lim) | 565333  511087  2177 |
| **Google Scholar** | search from cross references | 20 |
| Total Literatures Collected | | **28261** |

### Table A2. PICO characteristics of review question

| **PICO** | **Characteristics of Review Questions** |
| --- | --- |
| Population | Adults (over 18 years) with suspected primary hypertension |
| Target condition | Hypertension |
| Study design | Cross-sectional studies, diagnostic accuracy observational cohort studies, SRs of observational cohort |
| Intervention | Blood pressure measurement or monitoring by Digital bllod pressure Monitor |
| Comparison | Standard blood pressure monitoring by mercury sphygmomanometer |
| Outcome  Meta-analysis | Diagnostic odds ratio  Pooled Diagnostic odds ratio, proportion of true positive, Sensitivity and Specificity |

**Table A3. QUADAS-2 Evaluation Form for systematic review on diagnostic accuracy of various diagnostic tools to screen hypertension**

| **Domain/Question** | **Ostchega Y, et al. 2010** | **Vera-Cala LM, et al. 2011** | **Bhatt P, et al. 2016** | **Shahbabu B, et al. 2016** |
| --- | --- | --- | --- | --- |
|  | Yes/No/ Unclear | Yes/No/ Unclear | Yes/No/ Unclear | Yes/No/ Unclear |
| **Patient Selection** |  |  |  |  |
| Was a consecutive or random sample of patients enrolled? | No | Yes | No | Yes |
| Was a case–control design avoided? | No | Yes | No | Yes |
| Did the study avoid inappropriate exclusions? | No | Unclear | Unclear | Yes |
| **Index Test** |  |  |  |  |
| Were the index test results interpreted without knowledge of the results of the reference standard? | Yes | Yes | Yes | Yes |
| If a threshold was used, was it pre‐specified? | Yes | Yes | Yes | Yes |
| **Reference Standard** |  |  |  |  |
| Is the reference standard likely to correctly classify the target condition? | Unclear | Yes | Yes | Yes |
| Were the reference standard results interpreted without knowledge of the results of the index test? | Unclear | Yes | Yes | Yes |
| **Flow and Timing** |  |  |  |  |
| Was there an appropriate interval between index tests and reference standard? | Unclear | Unclear | Yes | Yes |
| Did all patients receive a reference standard? | Yes | No | Yes | Yes |
| Did all patients receive the same reference standard? | No | No | Yes | Yes |
| Were all patients included in the analysis? | No | No | Yes | Yes |

**Table A4. PRISMA 2020 for Abstracts Checklist**

| **Section and Topic** | **Item #** | **Checklist item** | **Reported (Yes/No)** |
| --- | --- | --- | --- |
| **TITLE** | | | Yes |
| Title | 1 | Identify the report as a systematic review. |  |
| **BACKGROUND** | | |  |
| Objectives | 2 | Provide an explicit statement of the main objective(s) or question(s) the review addresses. | Yes |
| **METHODS** | | |  |
| Eligibility criteria | 3 | Specify the inclusion and exclusion criteria for the review. | Yes |
| Information sources | 4 | Specify the information sources (e.g. databases, registers) used to identify studies and the date when each was last searched. | Yes |
| Risk of bias | 5 | Specify the methods used to assess risk of bias in the included studies. | Yes |
| Synthesis of results | 6 | Specify the methods used to present and synthesise results. | Yes |
| **RESULTS** | | |  |
| Included studies | 7 | Give the total number of included studies and participants and summarise relevant characteristics of studies. | Yes |
| Synthesis of results | 8 | Present results for main outcomes, preferably indicating the number of included studies and participants for each. If meta-analysis was done, report the summary estimate and confidence/credible interval. If comparing groups, indicate the direction of the effect (i.e. which group is favoured). | Yes |
| **DISCUSSION** | | |  |
| Limitations of evidence | 9 | Provide a brief summary of the limitations of the evidence included in the review (e.g. study risk of bias, inconsistency and imprecision). | Yes |
| Interpretation | 10 | Provide a general interpretation of the results and important implications. | Yes |
| **OTHER** | | |  |
| Funding | 11 | Specify the primary source of funding for the review. | Yes |
| Registration | 12 | Provide the register name and registration number. | Yes |

*From:*  Page MJ, McKenzie JE, Bossuyt PM, Boutron I, Hoffmann TC, Mulrow CD, et al. The PRISMA 2020 statement: an updated guideline for reporting systematic reviews. BMJ 2021;372:n71. doi: 10.1136/bmj.n71

**Table A5. PRISMA 2020 Checklist**

| **Section and Topic** | **Item #** | **Checklist item** | **Location where item is reported** |
| --- | --- | --- | --- |
| **TITLE** | | | Page 1 |
| Title | 1 | Identify the report as a systematic review. |  |
| **ABSTRACT** | | |  |
| Abstract | 2 | See the PRISMA 2020 for Abstracts checklist. | Page 2 |
| **INTRODUCTION** | | |  |
| Rationale | 3 | Describe the rationale for the review in the context of existing knowledge. | Page 3 |
| Objectives | 4 | Provide an explicit statement of the objective(s) or question(s) the review addresses. | Page 3 |
| **METHODS** | | |  |
| Eligibility criteria | 5 | Specify the inclusion and exclusion criteria for the review and how studies were grouped for the syntheses. | Page 4 |
| Information sources | 6 | Specify all databases, registers, websites, organisations, reference lists and other sources searched or consulted to identify studies. Specify the date when each source was last searched or consulted. | Page 4 |
| Search strategy | 7 | Present the full search strategies for all databases, registers and websites, including any filters and limits used. | Page 4 |
| Selection process | 8 | Specify the methods used to decide whether a study met the inclusion criteria of the review, including how many reviewers screened each record and each report retrieved, whether they worked independently, and if applicable, details of automation tools used in the process. | Page 4-5 |
| Data collection process | 9 | Specify the methods used to collect data from reports, including how many reviewers collected data from each report, whether they worked independently, any processes for obtaining or confirming data from study investigators, and if applicable, details of automation tools used in the process. | Page 5 |
| Data items | 10a | List and define all outcomes for which data were sought. Specify whether all results that were compatible with each outcome domain in each study were sought (e.g. for all measures, time points, analyses), and if not, the methods used to decide which results to collect. | Page 5 |
|  | 10b | List and define all other variables for which data were sought (e.g. participant and intervention characteristics, funding sources). Describe any assumptions made about any missing or unclear information. | NA |
| Study risk of bias assessment | 11 | Specify the methods used to assess risk of bias in the included studies, including details of the tool(s) used, how many reviewers assessed each study and whether they worked independently, and if applicable, details of automation tools used in the process. | Page 5 |
| Effect measures | 12 | Specify for each outcome the effect measure(s) (e.g. risk ratio, mean difference) used in the synthesis or presentation of results. | NA |
| Synthesis methods | 13a | Describe the processes used to decide which studies were eligible for each synthesis (e.g. tabulating the study intervention characteristics and comparing against the planned groups for each synthesis (item #5)). | Page 5 |
|  | 13b | Describe any methods required to prepare the data for presentation or synthesis, such as handling of missing summary statistics, or data conversions. | NA |
|  | 13c | Describe any methods used to tabulate or visually display results of individual studies and syntheses. | NA |
|  | 13d | Describe any methods used to synthesize results and provide a rationale for the choice(s). If meta-analysis was performed, describe the model(s), method(s) to identify the presence and extent of statistical heterogeneity, and software package(s) used. | Page 5 |
|  | 13e | Describe any methods used to explore possible causes of heterogeneity among study results (e.g. subgroup analysis, meta-regression). | Page 5 |
|  | 13f | Describe any sensitivity analyses conducted to assess robustness of the synthesized results. | Page 5 |
| Reporting bias assessment | 14 | Describe any methods used to assess risk of bias due to missing results in a synthesis (arising from reporting biases). | NA |
| Certainty assessment | 15 | Describe any methods used to assess certainty (or confidence) in the body of evidence for an outcome. | NA |
| **RESULTS** | | |  |
| Study selection | 16a | Describe the results of the search and selection process, from the number of records identified in the search to the number of studies included in the review, ideally using a flow diagram. | Page 6; Figrue-1 |
|  | 16b | Cite studies that might appear to meet the inclusion criteria, but which were excluded, and explain why they were excluded. | Page 6 |
| Study characteristics | 17 | Cite each included study and present its characteristics. | Page 6 |
| Risk of bias in studies | 18 | Present assessments of risk of bias for each included study. | Page 7; Table A3 |
| Results of individual studies | 19 | For all outcomes, present, for each study: (a) summary statistics for each group (where appropriate) and (b) an effect estimate and its precision (e.g. confidence/credible interval), ideally using structured tables or plots. | Page 6; Table-1 |
| Results of syntheses | 20a | For each synthesis, briefly summarise the characteristics and risk of bias among contributing studies. | Page 6; Table-2 |
|  | 20b | Present results of all statistical syntheses conducted. If meta-analysis was done, present for each the summary estimate and its precision (e.g. confidence/credible interval) and measures of statistical heterogeneity. If comparing groups, describe the direction of the effect. | Page 7; Table-2 |
|  | 20c | Present results of all investigations of possible causes of heterogeneity among study results. | Page 7; Figure 3-5 |
|  | 20d | Present results of all sensitivity analyses conducted to assess the robustness of the synthesized results. | NA |
| Reporting biases | 21 | Present assessments of risk of bias due to missing results (arising from reporting biases) for each synthesis assessed. | NA |
| Certainty of evidence | 22 | Present assessments of certainty (or confidence) in the body of evidence for each outcome assessed. | NA |
| **DISCUSSION** | | |  |
| Discussion | 23a | Provide a general interpretation of the results in the context of other evidence. | Page-7 & Page-8 |
|  | 23b | Discuss any limitations of the evidence included in the review. | Page-9 |
|  | 23c | Discuss any limitations of the review processes used. | NA |
|  | 23d | Discuss implications of the results for practice, policy, and future research. | Page-9 |
| **OTHER INFORMATION** | | |  |
| Registration and protocol | 24a | Provide registration information for the review, including register name and registration number, or state that the review was not registered. | Page-3 |
|  | 24b | Indicate where the review protocol can be accessed, or state that a protocol was not prepared. | Yes |
|  | 24c | Describe and explain any amendments to information provided at registration or in the protocol. | NA |
| Support | 25 | Describe sources of financial or non-financial support for the review, and the role of the funders or sponsors in the review. | Page-1 |
| Competing interests | 26 | Declare any competing interests of review authors. | Page-1 |
| Availability of data, code and other materials | 27 | Report which of the following are publicly available and where they can be found: template data collection forms; data extracted from included studies; data used for all analyses; analytic code; any other materials used in the review. | Page-1 |

*From:*  Page MJ, McKenzie JE, Bossuyt PM, Boutron I, Hoffmann TC, Mulrow CD, et al. The PRISMA 2020 statement: an updated guideline for reporting systematic reviews. BMJ 2021;372:n71. doi: 10.1136/bmj.n71
